# Clinical analysis of hypereosinophilic syndrome first presenting with asthma-like symptoms

**DOI:** 10.1080/07853890.2021.2014555

**Published:** 2021-12-22

**Authors:** Xuan Wei, Xiaofeng Li, Zuyou Wei, Hui Zhang, Jiehua Deng, Suke Xing, Jianquan Zhang

**Affiliations:** a Department of Respiratory and Critical Care Medicine, The First Affiliated Hospital of Guangxi Medical University, Nanning, Guangxi, China; b Department of Respiratory Medicine, The Eighth Affiliated Hospital, Sun Yat Sen University, Shenzhen, Guangdong, China

**Keywords:** Eosinophils, idiopathic, parasitic infection, glucocorticoid

## Abstract

**Introduction:**

Clinical manifestations of hypereosinophilic syndrome (HES) are diverse. This study aimed to summarise these clinical characteristics with asthma-like onset as the first symptom, and compare these characteristics and treatment strategies between idiopathic and parasitic HES.

**Materials and methods:**

We retrospectively analysed 36 HES patients with asthma-like symptoms as the first episode, between January 2013 and October 2019. Data of patients with HES of an unknown cause (idiopathic HES) and parasitic infection (parasite HES) were analysed.

**Results:**

The idiopathic and parasite HES groups included 16 and 20 patients, respectively, with more males in the parasite HES group (*p* < .05). Wheezing and dry rales was the most common symptom and signs, with no significant differences in symptoms and signs between the groups. The most often misdiagnosed disease was bronchial asthma. The peripheral blood eosinophil count was significantly increased compared with normal counts in both groups (*p* > .05). Abnormal pulmonary function is mainly manifested as obstructive ventilatory disorder and mixed ventilatory disorder. Chest computed tomography showed extensive ground-glass exudation, patches, consolidation, nodules, and pleural effusion. Histopathological examination showed eosinophilic infiltration without vasculitis or granuloma. Glucocorticoids had a significant therapeutic effect, and the parasite HES group required combined deworming drugs. The duration of corticosteroids therapy in the idiopathic HES group was significantly longer than that in the parasite HES group (*p* < .05). The overall prognosis was good, and 81.25% of the patients were clinically cured in the parasite HES group; however, relapse occurred easily in the idiopathic HES group.

**Conclusions:**

Asthma-like symptoms, obstructive ventilatory disorder or positive bronchial dilation test, and poor response to inhaled corticosteroids are not necessarily indicative of refractory asthma; HES should be considered. The clinical characteristics of HES of different aetiologies are similar. Systemic corticosteroid therapy is preferred for idiopathic and parasitic infections. Idiopathic HES is treated with prolonged corticosteroids and relapses easily.

**Key Messages**
- Asthma-like symptoms, obstructive ventilatory disorder or positive bronchial dilation tests, and poor responses to inhaled corticosteroids are not necessarily indicative of refractory asthma, and hypereosinophilic syndrome should be considered.
- The clinical characteristics of hypereosinophilic syndrome of different aetiologies are similar, and systemic glucocorticoid therapy is preferred for both idiopathic and parasitic infections.
- Idiopathic hypereosinophilic syndrome is treated with prolonged corticosteroids and relapses easily.

## Introduction

Hypereosinophilic syndrome (HES) is characterised by persistently elevated levels of eosinophils (EOS), their infiltration into various tissues and organs, and the emergence of corresponding clinical symptoms and signs; the clinical manifestations are complex and diverse. In 2011, the Working Conference on Eosinophil Disorders and Syndromes referred to any hypereosinophilia associated with organ damage as HES, clearly defining idiopathic hypereosinophilic syndrome (HES_US_), primary hypereosinophilic syndrome (HES_N_), secondary hypereosinophilic syndrome (HES_R_), and other conditions and syndromes [1]. Because HES_US_ is diagnosed by exclusion, it is difficult to rapidly and effectively diagnose in a clinical setting. One of the most common causes of HES_R_ is parasite infection, which is often ignored and may lead to multiple organ failure [2,3].

Eosinophil-related respiratory diseases are a global concern, and patients can have a variety of clinical characteristics. EOS located in the airway can cause chronic inflammation (e.g. asthma and COPD), while EOS can also significantly increase in peripheral blood EOS (even as one of the diagnostic criteria, such as allergic bronchopulmonary aspergillosis [ABPA] and eosinophilic granulomatosis with polyangiitis [EGPA]) [4]. Although HES often involves the respiratory system, eosinophilic pneumonia and pleural effusion were the most common in previous reports [5]. However, HES with airway hyperresponsiveness (AHR), such as repeated wheezing attacks as first symptoms, and with obstructed but reversible pulmonary functions, is rare and garners little attention. Patients are often misdiagnosed as refractory bronchial asthma, resulting in long-term misdiagnosis and mistreatment, and even death. To improve our understanding of the clinical diversity of HES, we aimed to retrospectively analyse the clinical features of HES with asthma-like symptoms as the initial symptom, and compare the differences in clinical features between idiopathic and parasitic HES.

## Materials and methods

### Clinical data

We retrospectively analysed patients with HES who had initially presented with asthma-like symptoms between January 1, 2013 and October 1, 2019 at our institution. The basic condition, clinical data, and follow-up data of the patients were collected and statistically analysed. According to the strict regulations on a retrospective study of the Ethics Committee of the first affiliated Hospital of Guangxi Medical University, written informed consent was obtained in all cases (signed by the patient or their immediate family) prior to the study, and the approved ethics was 2021.KY-E-091.

### Inclusion criteria

We included patients who met the following diagnostic criteria [1]: EOS count >1.5 × 10^9^/L on two blood examinations (interval ≥1 month); ≥two organs damaged and/or dysfunctional, attributed to tissue hypereosinophilia; patients with systematic examination results, except for organ damage or dysfunction caused by other diseases or conditions; negative *ETV6-PDGFRβ, FIP1L1-PDGFRα, FGFR1*, and *JAK2* gene tests; and haematologist consultation records excluding HES_N_. The asthma-like symptoms include paroxysmal cough or wheezing. The patients with unknown causes were assigned to the idiopathic HES group, while those with clear parasitic infection were assigned to the parasite HES group.

### Statistical analyses

Statistical analyses were performed using SPSS software (Windows version 25.0; SPSS Inc., Chicago, IL, USA). Continuous variables are represented as medians (interquartile range), categorical variables are expressed as counts (%), measurement data used rank-sum test (Wilcoxon rank-sum test or signed rank-sum test) for comparison between groups, and count data were analysed using the chi-square test or Fisher’s exact test for comparison between groups. Statistical significance was defined as a *P*-value of <.05.

## Results

### General data and clinical features

A total of 44 idiopathic HES and 34 parasitic HES cases were identified, of which 36 patients with asthma-like symptoms as the initial symptom were included in this study (16 [36.36%] patients in the idiopathic HES group and 20 [58.82%] in the parasite HES group). There was no statistical difference in age of onset between the two groups. There were more males than females in the parasite HES group (*p* < .05, Table 1), with a male-to-female ratio of 17:3. The median time from symptom onset to diagnosis was 2 (1–10.5) months in the idiopathic HES group and 6 (1–12) months in the parasite HES group. There were no significant differences in the symptoms and signs between the two groups. Patients were misdiagnosed with bronchial asthma, pneumonia, chronic obstructive pulmonary disease (COPD), lung neoplasms, tuberculosis, and pulmonary embolism (Table 1).

**Table 1.** General data and clinical features of patients in idiopathic HES and parasite HES groups.

Clinical features	Idiopathic HES group (*n* = 16)	Parasite HES group (*n* = 20)	*P* value
Age (y)	46.5 (35.25–51.00)	54.50 (35.75–64.75)	.067
Male	8 (50%)	17 (85%)	.034*
Female	8 (50%)	3 (15%)	
Wheeze	14 (87.50%)	20 (100%)	.190
Cough	16 (100%)	17 (85%)	.238
Expectoration	11 (68.75%)	12 (60%)	.731
Chest tightness	4 (25%)	6 (30%)	1.000
Chest pain	3 (18.75%)	6 (30%)	.700
Erythra	4 (25%)	1 (5%)	.149
Dry rale	14 (87.50%)	16 (80%)	.672
Moist rales	3 (18.75%)	3 (15%)	1.000
Misdiagnosis			
Bronchial asthma	12 (75%)	16 (80%)	1.000
Pneumonia	5 (31.25%)	8 (40%)	.731
Chronic obstructive pulmonary disease	0 (0)	4 (20%)	.113
Lung neoplasms	1 (6.25%)	1 (5%)	1.000
Pulmonary tuberculosis or tuberculous pleurisy	0 (0)	2 (10%)	.492
Pulmonary embolism	3 (18.75%)	0 (0)	.078
Median time from onset of symptoms to diagnosis (M)	2 (1–10.5)	6 (1–12)	.328

Data are expressed as the number and percentage or median (interquartile range).

y, year; M, month; idiopathic HES group, patients with HES of an unknown cause; parasite HES group, patients with parasitic infection. *p < .05 is statistically significant.

All cases showed multiple systemic involvements. Vascular and skin involvement were found in five cases, respectively. Cardiovascular examination results of electrocardiography (ECG) and echocardiography were abnormal in 10 cases. Five patients had abnormal digestive system examination results. Lymph node enlargement was observed in eight patients. In the parasite HES group, parasite eggs in the stool were found in all patients.

### Laboratory results, pulmonary function, and imaging

Despite the elevated or abnormal results, there were no significant differences in the peripheral blood EOS count, erythrocyte sedimentation rate, C-reactive protein, immunoglobulin E (IgE), and myocardial enzyme levels between the two groups (Table 2). Tumour markers, immunoglobulins, and autoantibodies were normal in all cases, including anti-neutrophil cytoplasmic antibodies (ANCA).

**Table 2.** Comparison of laboratory examination results between idiopathic HES and parasite HES groups.

Laboratory examination	Idiopathic HES group (*n* = 16)	Parasite HES group (*n* = 20)	*P* value
White blood cell (10^9^/L)	11.11 (7.73–15.56)	10.04 (8.41–17.53)	.799
Eosinophil (10^9^/L)	2.37 (1.76–4.54)	2.13 (1.84–6.17)	.633
Partial pressure of oxygen (mmHg)	77.90 (67.08–83.18)^a^	76.20 (70.75–78.70)^b^	.763
Partial pressure of carbon dioxide (mmHg)	36.55 (32.6–40.03)^a^	40 (36.65–41.9)^b^	.025*
Immunoglobulin E (IU/mL)	322.4 (131.4–750.4)^c^	489.8 (24.8–1000.7)^d^	.855
Erythrocyte sedimentation rate (>20mm/H)	7 (43.75%)	7 (35%)	.734
C-reactive protein(>10mg/L)	6 (37.5%)	6 (30%)	.729
Lactate dehydrogenase (>245 U/L)	9 (56.25%)	11 (55%)	1.000

^a^*n* = 10; ^b^*n* = 17; ^c^*n* = 5; ^d^*n* = 6.

Reference values: Leucocyte: 3.5–9.5 × 10^9^/L; Eosinophil: 0.02–0.52 × 10^9^/L.

Immunoglobulin E: 0–100 U/mL; The reference critical values of the other indicators are all values in parentheses. EOS, eosinophils; idiopathic HES group, patients with HES of an unknown cause; parasite HES group, patients with parasitic infection.

*p < .05, statistically significant. Data are expressed as the number and percentage or median (interquartile range).

**Figure 1.**
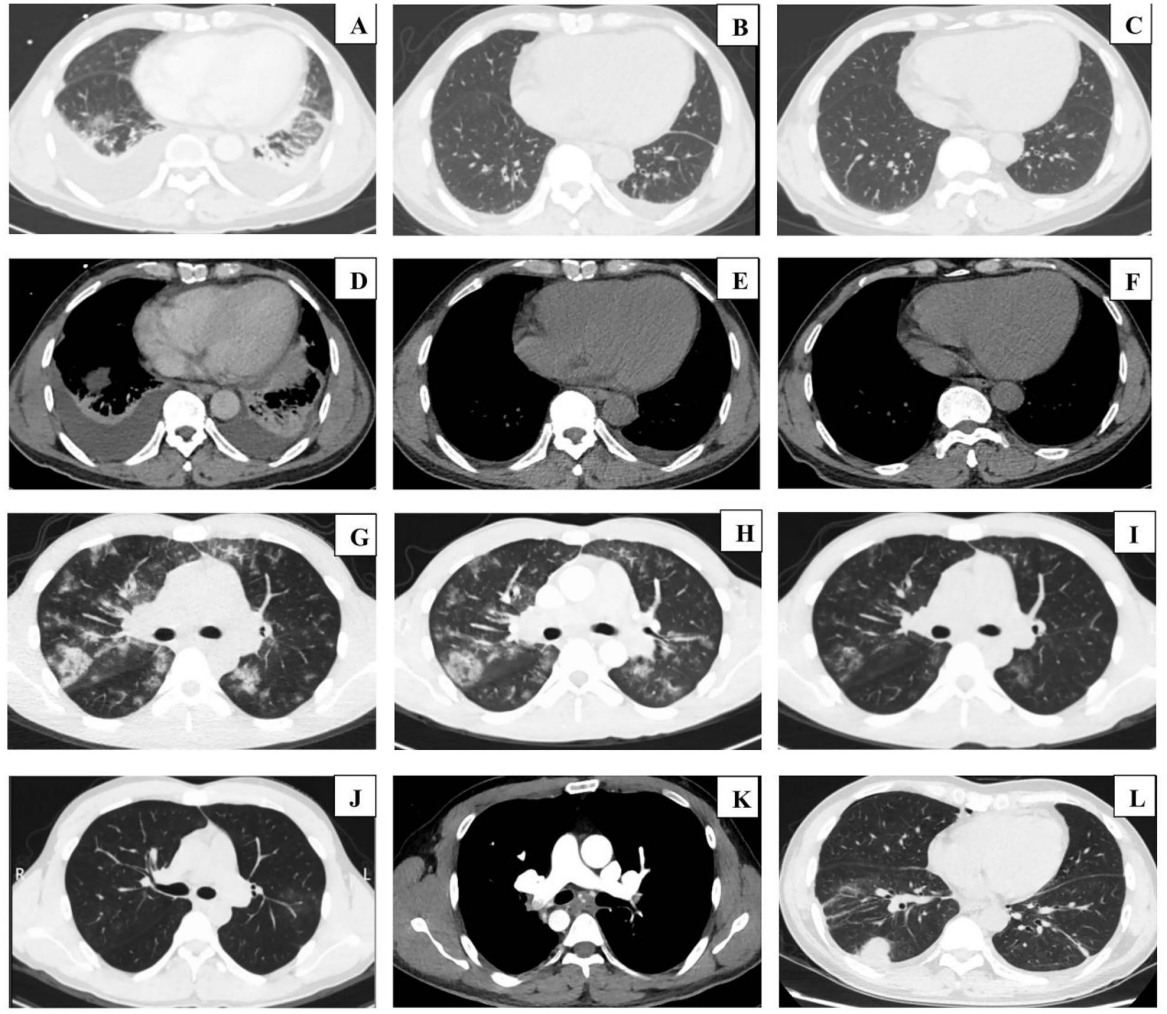
Chest CT showing patches and consolidation, and bilateral pleural effusion. The lesion disappears after applying glucocorticoid combined with deworming drugs. (A,D) before treatment, (B,E) after 7 days of treatment, (C,F) after 7 weeks of treatment (patients from the parasite HES group). Chest CT showing extensive ground-glass exudation and patches shadow in both lungs, the lesion is gradually absorbed after glucocorticoid therapy: (G) before treatment; (H) after 2 days of treatment; (I) after 5 days of treatment; (J) after 9 weeks of treatment (patients from the idiopathic HES group). Chest CT shows pulmonary embolism (K) and nodule (L). CT, computed tomography; HES, hypereosinophilic syndrome.

One patient had pericardial effusion. Colour ultrasound showed ascites, hepatosplenomegaly, superficial lymph node enlargement, and superficial venous thrombosis. Thirty-one patients completed the pulmonary function examination, of whom eight showed normal results (because of the serious condition, they all completed the examination after treatment). There were 23 patients with abnormal pulmonary function, of whom eight were positive for bronchial dilation test (BDT). There were no significant differences in pulmonary function between the two groups. High-resolution chest computed tomography (HRCT) was performed in all cases, and the results showed extensive ground-glass exudation, patches, consolidation, nodules, pulmonary embolism, and pleural effusion (Figure 1). Ground-glass exudation was more commonly observed in the idiopathic HES group than in the parasite HES group (*p* < .05) (Table 3).

**Table 3.** Comparison of imaging examination results between idiopathic HES and parasite HES groups.

	Idiopathic HES group (*n* = 16)	Parasite HES group (*n* = 20)	*P* value
Abnormal cardiac ultrasound	0 (0)	1 (5%)	1.000
Peritoneal effusion	0 (0)	1 (5%)	1.000
Hepatomegaly/splenomegaly	0 (0)	2 (10%)	.492
Superficial lymph node enlargement	2 (12.5%)	2 (10%)	1.000
Peripheral venous thrombosis	2 (12.5%)	0 (0)	.190
Abnormal pulmonary function	10/14 (71.42%)	13/17 (76.41%)	1.000
Obstructive ventilatory disorder	5 (35.71%)	10 (58.82%)	.285
Mixed ventilatory disorder	5 (35.71%)	3 (17.65%)	.412
Diffusion disorder	6 (42.86%)	5 (29.41%)	.447
Bronchial dilation test positive	3 (21.43%)	5 (29.41%)	.698
Chest CT or CTPA			–
Ground-glass shadow	9 (56.25%)	2 (10%)	.004*
Patches or solid shadows	8 (50%)	11 (55%)	1.000
Mass, nodular shadow	3 (18.75%)	5 (25%)	.709
Pleural effusion	5 (31.25%)	6 (30%)	1.000
Pericardial effusion	0 (0)	1 (5%)	1.000
Mediastinal lymph node enlargement	1 (6.25%)	3 (15%)	.613
Pulmonary embolism	3 (18.75%)	0 (0)	.078

Idiopathic HES group, patients with HES of an unknown cause; parasite HES group, patients with parasitic infection. CT, computed tomography; CTPA, computed tomography pulmonary angiogram.

*p < .05, statistically significant. Data are expressed as the number and percentage.

### Cytology and histopathology

**Figure 2.**
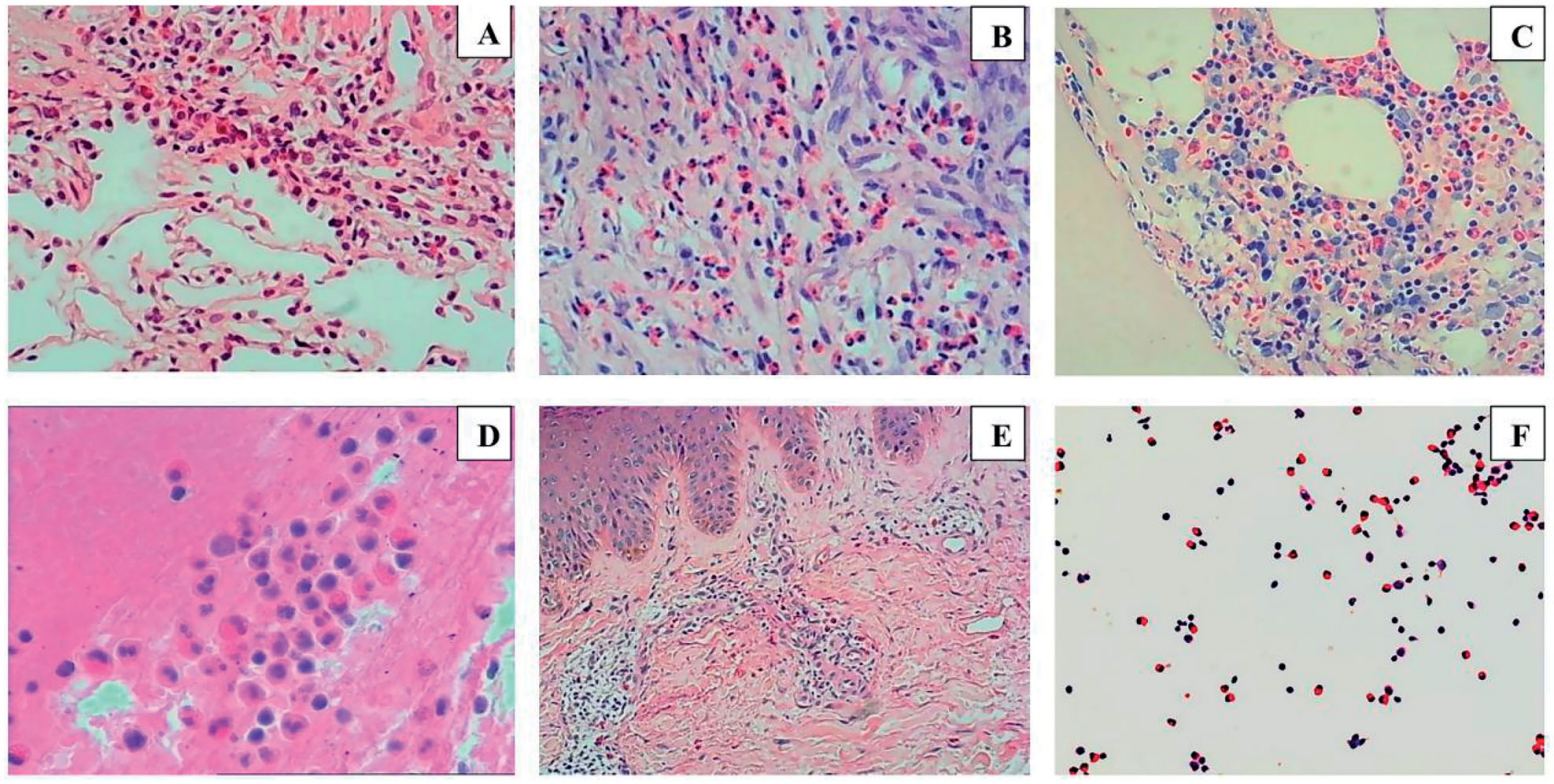
Pathological examination image (HE staining). (A) Infiltration of eosinophils and lymphocytes in lung interstitium (magnification: ×200). (B) Pleural tissue shows many eosinophils infiltrated in the fibrous connective tissue (magnification: ×200). (C) Pronounced eosinophil infiltration in bone marrow tissue (magnification: ×200). (D) EOS infiltration is seen in mediastinal lymph nodes (magnification: ×400). (E) EOS infiltration in the skin tissue of the right lower limb (magnification: ×100). (F) EOS in pleural effusion smears (×200). HE, haematoxylin and eosin; EOS, eosinophils.

All patients were examined by bone marrow cytology or bone marrow biopsy. *ETV6-PDGFR β* and *FIP1L1-PDGFR α* gene results, and *FGFR1* and *JAK2* genes results, were negative in five patients. Pathological examination of the bone marrow tissue confirmed greater infiltration of EOS, and smears showed an EOS proportion of >20% in 19 cases. Tissue biopsies from the lung, pleura, lymph node, and skin tissues all showed high infiltration of EOS, and no fibrinous necrotising vasculitis or necrotising granuloma (Figure 2). In addition, many EOS were found in bronchoalveolar lavage fluid (BALF) smears and pleural effusion smears. Positive EOS in pleural fluid is defined as a pleural effusion that contains ≥10% eosinophils [6]. Positive EOS in bronchoalveolar lavage fluid is defined as containing ≥5% eosinophils [7] (Table 4).

**Table 4.** Comparison of pathological examination results between idiopathic HES and parasite HES groups.

	Idiopathic HES group (*n* = 16)	Parasite HES group (*n* = 20)	*P* value
EOS ratio of bone marrow smea*r* ≥ 20%	10 (62.5%)	9 (45%)	.335
EOS infiltrate in bone marrow biopsy	12/15 (80%)	6/9 (66.67%)	.635
Positive EOS in sputum smear	0 (0)	1/1 (100%)	–
Positive EOS in pleural fluid	1/2 (50%)	1/3 (33.33%)	1.000
Positive EOS in bronchoalveolar lavage fluid	2/9 (22.22%)	5/11 (45.45%)	.374
EOS infiltrate lung and pleura	2/5 (40%)	4/9 (44.44%)	1.000
EOS infiltrate lymph nodes	1/1 (100%)	0 (0)	–
EOS infiltrate skin tissue	0 (0)	1/1 (100%)	–

EOS, eosinophils; idiopathic HES group, patients with HES of an unknown cause; parasite HES group, patients with parasitic infection.

*p < .05, statistically significant. Data are expressed as the number and percentage.

### Treatment and outcomes

Fourteen patients in the idiopathic HES group were administered corticosteroids, with an initial dose of prednisone 20–80 mg/day. In the parasite HES group, 16 patients were administered glucocorticoids combined with praziquantel or albendazole (initial dose of prednisone was 10–80 mg/day; the total dose of praziquantel was 150 mg/kg for an average of 3 days, and albendazole was 10–20 mg/kg/day for 7 days). After 1–4 weeks of steroid treatment, symptoms and related indicators were improved, and the differences before and after treatment were statistically significant (*p* < .05) (Table 5). The remaining two patients in the idiopathic HES group (ICS only), and four patients in the parasite HES group (deworming only) were not treated with corticosteroids and had poor responses.

**Table 5.** Comparison of laboratory examination and pulmonary function before and after treatment.

	Pretherapy	Post-treatment	*P* value
Idiopathic HES group (*n* = 14)			
White blood cell (10^9^/L)	10.62 (7.19–17.18)	12.22 (8.89–15.44)	1.000
Eosinophil (10^9^/L)	2.37 (1.85–5.01)	0.39 (0.07–0.95)	.002*
Arterial oxygen pressure (mmHg)^a^	68.75 (63.30–84.50)	93.65 (74.43–106.00)	.031*
Arterial carbon dioxide partial pressure (mmHg)^a^	33.5 (31.25–40.13)	36.9 (34.18–39.18)	.687
FEV1 (%)^b^	38.40 (28.1–67.90)	63.80 (48.80–91.60)	.016*
FVC (%)^b^	61.70 (39.60–83.30)	87.0 (76.60–100.70)	.016*
FEV1/FVC (%)^b^	68.94 (52.04–79.42)	71.50 (61.60–77.67)	.453
Parasite HES group (*n* = 16)			
White blood cell (10^9^/L)	9.59 (7.5–18.81)	9.31 (7.58–13.48)	.804
Eosinophil (10^9^/L)	2.02 (1.81–5.48)	0.62 (0.18–0.96)	.001*
Partial pressure of oxygen (mmHg)^c^	66.4 (64.5–68.3)	75.7 (70.4–81)	–
Partial pressure of carbon dioxide (mmHg)^c^	39.1 (44.6–33.6)	43.45 (48.7–38.2)	–
FEV1 (%)^d^	50.40 (36.35–67.10)	80.50 (73.50–99.0)	.039*
FVC (%)^d^	76.90 (68.95–87.55)	101.0 (90.15–109.80)	.180
FEV1/FVC (%)^d^	53.90 (42.90–63.03)	69.7 (58.55–79.85)	.289

^a^*n* = 6; ^b^*n* = 7; ^c^*n* = 2; ^d^*n* = 9. Data are expressed as median (interquartile range).

FEV1: forced expiratory volume in one second; FVC: forced vital capacity; idiopathic HES group, patients with HES of an unknown cause (steroid therapy for 1–4 weeks); parasite HES group, patients with parasitic infection (steroid combined with deworming therapy for 1–4 weeks).

*p < .05, statistically significant.

According to the patient’s condition, glucocorticoids were gradually decreased and discontinued, and patients were followed up for 6–12 months. The duration of glucocorticoid therapy in the idiopathic HES group was significantly longer than that in the parasite HES group (4 [2.75–11.5] vs. 2.25 [1–3] months, *p* < .05). The overall prognosis was good, and 81.25% of the patients were clinically cured in the parasite HES group; however, relapse occurred easily in the idiopathic HES group. A comparison of outcomes between the idiopathic HES and parasite HES groups showed a significant difference in prognosis between the two groups (*p* < .05) (Table 6).

**Table 6.** Comparison of glucocorticoid use time and outcome between idiopathic HES and parasite HES groups.

	Idiopathic HES group (*n* = 14)	Parasite HES group (*n* = 16)	*P* value
Duration of glucocorticoid use (M)	4 (2.75–11.5)	2.25 (1–3)	.007*
Clinical cure	5 (35.71%)	13 (81.25%)	.024*
No recurrence	2 (40%)	12 (92.31%)	.044*
Recrudesce	3 (60%)	1 (7.69%)	.044*
Re-clinical cure	1 (33.33%)	0 (0)	1.000
Re-improvement	1 (33.33%)	1 (100%)	1.000
Re-under treatment	1 (33.33%)	0 (0)	1.000
Improvement	9 (64.29%)	2 (12.5%)	.007*
Death	0 (0)	1 (6.25%)	1.000

*p < .05, statistically significant. Data are expressed as the number and percentage or median (interquartile range).Clinical cure: the symptoms and signs of the patient disappeared, there is no abnormality in laboratory and imaging examination, the standard of drug withdrawal was reached and the drug stopped, and there was a follow-up for 6–12 months without relapse; recurrence: the recurrence of the same or similar clinical manifestations during the follow-up after drug withdrawal; improvement: the condition is relieved after treatment, the laboratory and imaging examinations are better than before, and the drug dose has been gradually reduced but did not meet the drug withdrawal standard; the patient did not return to the hospital as required in the follow-up stage; death: the cause of death was attributed to hypereosinophilic syndrome.

Idiopathic HES group, patients with HES of an unknown cause; parasite HES group, patients with parasitic infection; M, months.

## Discussion

HES results in continuously elevated EOS infiltration into systemic tissues, causing inflammatory damage and associated complex clinical features. Involvement of the respiratory system is characterised by common symptoms as well as obvious AHR, such as paroxysmal cough or wheezing (asthma-like symptoms). There is little evidence on the association between asthma and HES [8]; therefore, it is easy to ignore the manifestations of extrapulmonary involvement and misdiagnose eosinophilic pneumonia or bronchial asthma due to prolonged wheezing symptoms. Patients with paroxysmal wheezing or cough who were diagnosed with numerous acute attacks of bronchial asthma were finally diagnosed with HES of different causes [9–12], suggesting that asthma-like symptoms may be a prominent manifestation in some HES patients. When there is mild or no involvement of the extrapulmonary organs, the clinical manifestations are minor, and hospitalisation temporarily partially relieves the symptoms, it is easy to erroneously diagnose such patients with bronchial asthma. This can worsen the disease and delay treatment which, in severe cases, can lead to multiple organ failure or even death [13]. Therefore, it is pertinent to summarise the clinical characteristics of HES initially presenting with asthma-like symptoms, and analyse the characteristics and treatment of idiopathic and parasitic HES, to improve clinician understanding of the disease.

The most common cause of secondary HES is parasitic infection (predominantly male) [1,14]. In this study, the male-to-female ratio in the parasite HES group was 17:3, which may be related to male social activities and an unclean diet; however, the male-to-female ratio of idiopathic HES group patients was 1:1, which is consistent with the literature [15]. Among both groups, 74.19% of patients had an abnormal pulmonary function, of whom eight were positive for BDT. The remaining eight patients with normal pulmonary function were examined only after their wheezing symptoms were relieved. It is suggested that abnormal lung function due to pulmonary involvement from idiopathic or parasitic HES is reversible. Our research shows that there was no significant difference in the clinical manifestations, laboratory test results, and pulmonary function between the two groups; therefore, it is difficult to distinguish between parasite HES and idiopathic HES based on the aforementioned manifestations, which also appear very similar to those of bronchial asthma. In addition to an EOS count >1.5 × 10^9^/L, multiple organ dysfunction is also a major feature of HES. In this study, a rash, abnormal colour ultrasound, thrombosis, lymph node enlargement, chest CT, and abnormal pathological manifestations suggested that the blood, lungs, heart, gastrointestinal tract, lymph nodes, and skin were involved. Of course, malignant tumours and autoimmune diseases were excluded. The exclusion of secondary factors is the first step in the HES diagnostic process, and comprehensive personal history data collection and repeated parasitic testing are the keys for screening for parasitic infections.

Our study showed that bronchial asthma, pulmonary infection, and COPD were the most common misdiagnosed diseases; the experience of medical treatment in the idiopathic HES and parasite HES groups was similar, and there was no significant difference in the median time from the onset of symptoms to diagnosis. With asthma-like manifestations as the first symptom, obstructive ventilation dysfunction is completely reversible or irreversible and should be distinguished from bronchial asthma or refractory asthma. The eosinophil count in the peripheral blood of asthma was far less than 1.5 × 10^9^/L, without other organ infiltrates and ICS with good effect [16–18]. Therefore, persistently elevated levels of EOS, bone marrow infiltration of EOS, and systemic damage cannot be explained by asthma, as is crucial to make the final diagnosis. Middle-aged and elderly men with a long history of smoking have recurrent wheezing and obstructive ventilatory dysfunction, which should be distinguished from COPD. Pulmonary exudation can also be observed when acute exacerbation of COPD is complicated with infection but can be absorbed after anti-infection. Although the symptoms can be relieved after systemic steroid treatment, the pulmonary function cannot return to normal, which is obviously different from that in patients in our study. ABPA can also cause similar manifestations, due to the allergic reaction induced by Aspergillus spores inhaled into the respiratory tract, but ABPA does not cause EOS infiltration into the bone marrow, and the total serum IgE level is >1,000 IU/mL. Pulmonary imaging also shows characteristic lesions (tree bud sign, mucus thrombus, and central bronchiectasis) and Aspergillus can be cultured in BALF. When asthma-like symptoms are poorly controlled, it is necessary to determine whether other systems are damaged. Symptoms of EGPA (prodromal phase and/or infiltration of EOS phase) are also similar to those of HES observed in our study. It can also involve multiple systems, which makes it difficult to distinguish clinically. When the lungs were involved, the chest CT also showed ground-glass exudation, consolidation, and nodules. However, EGPA is essentially vasculitis. When it develops into vasculitis, there are obvious systemic symptoms such as fever, weight loss, fatigue, abnormal inflammatory and immune indices, positive autoantibodies including anti-neutrophil cytoplasmic antibody, and histopathological changes (small vessel necrotising vasculitis and granuloma accompanied by infiltration of EOS in surrounding tissues), which are important for the diagnosis of EGPA. In our study, all cases were negative for autoantibodies and showed no vasculitis or granuloma on histopathological examination, which was the key for excluding EGPA.

The underlying mechanism of wheezing caused by HES remains unclear. Studies have shown that EOS cationic protein and peroxidase released by EOS can destroy the integrity of the bronchial epithelium; induce the release of histamines by mast cells; and cause bronchial contraction, edoema, and airway remodelling [4]. In this study, many EOS and lymphocytes infiltrated the alveolar interstitium, and EOS were found in BALF and sputum smears, suggesting that EOS aggregate, activate, and release inflammatory mediators in the bronchial and pulmonary interstitium, leading to AHR. After treatment, the EOS count was decreased and wheezing symptoms disappeared in both groups, suggesting that chronic inflammation induced by EOS in the respiratory tract may be the cause of asthma-like symptoms in HES. In addition, degranulation of EOS and release of a variety of mediators can stimulate platelet activation and aggregation, and damage vascular endothelial cells, both of which promote thrombosis. The tissue factor stored by EOS is the main initiator of blood clotting [19–21]; therefore, thrombosis is the main manifestation of HES involving blood vessels [22–24]. In the idiopathic HES group, three patients had pulmonary artery involvement and two had peripheral vein involvement, which was misdiagnosed as simple thromboembolism. The anticoagulant effect is poor and life-threatening in severe cases. These characteristics suggest that clinicians should pay attention to the EOS count in patients with pulmonary or other vascular embolisms, especially in those with a recurrent embolism or poor anticoagulant response.

Regardless of the cause of HES, the purpose of therapy is to reduce the EOS count and EOS-mediated organ dysfunction. The main therapeutic options for HES patients can be divided into five groups: corticosteroids; cytotoxic agents; tyrosine kinase inhibitors (TKIs); monoclonal antibodies; and chemotherapy [25] .Corticosteroids can be used as first-line therapy in patients with strictly defined HES, and the recommended dose is prednisone 1 mg/kg/day [26] .With the control of symptoms and a decrease in EOS count <1.5 × 10^9^/L, the dose of prednisone can be gradually reduced [27]. However, secondary HES should be treated according to the primary aetiology, and individualised treatment should be provided for vital organ involvement. A combination of corticosteroids is recommended, starting with prednisone at 1 mg/kg/day [28]. In this study, the treatment efficacy in patients in both groups was significant. After 1–4 weeks of treatment, symptoms were relieved or disappeared, EOS count decreased, lung function recovered, and the differences before and after treatment were statistically significant. The duration of steroid therapy in the idiopathic HES group was significantly longer than that in the parasite HES group. The prognosis of the parasite HES group was good, and 81.25% of the patients were successfully treated. Among them, one patient had a deteriorated condition and subsequently died during the maintenance of steroid reduction, suggesting that the injury caused by a parasitic infection should not be ignored. The allergic state of the body is activated, and re-exposure to allergens is likely to lead to recurrence or deterioration. In the idiopathic HES group, it is easy to relapse, and steroid therapy is lengthy. Nine patients did not return to the hospital during the period of steroid reduction after disease control; therefore, the median time of steroid therapy should be longer.

**Figure 3.**
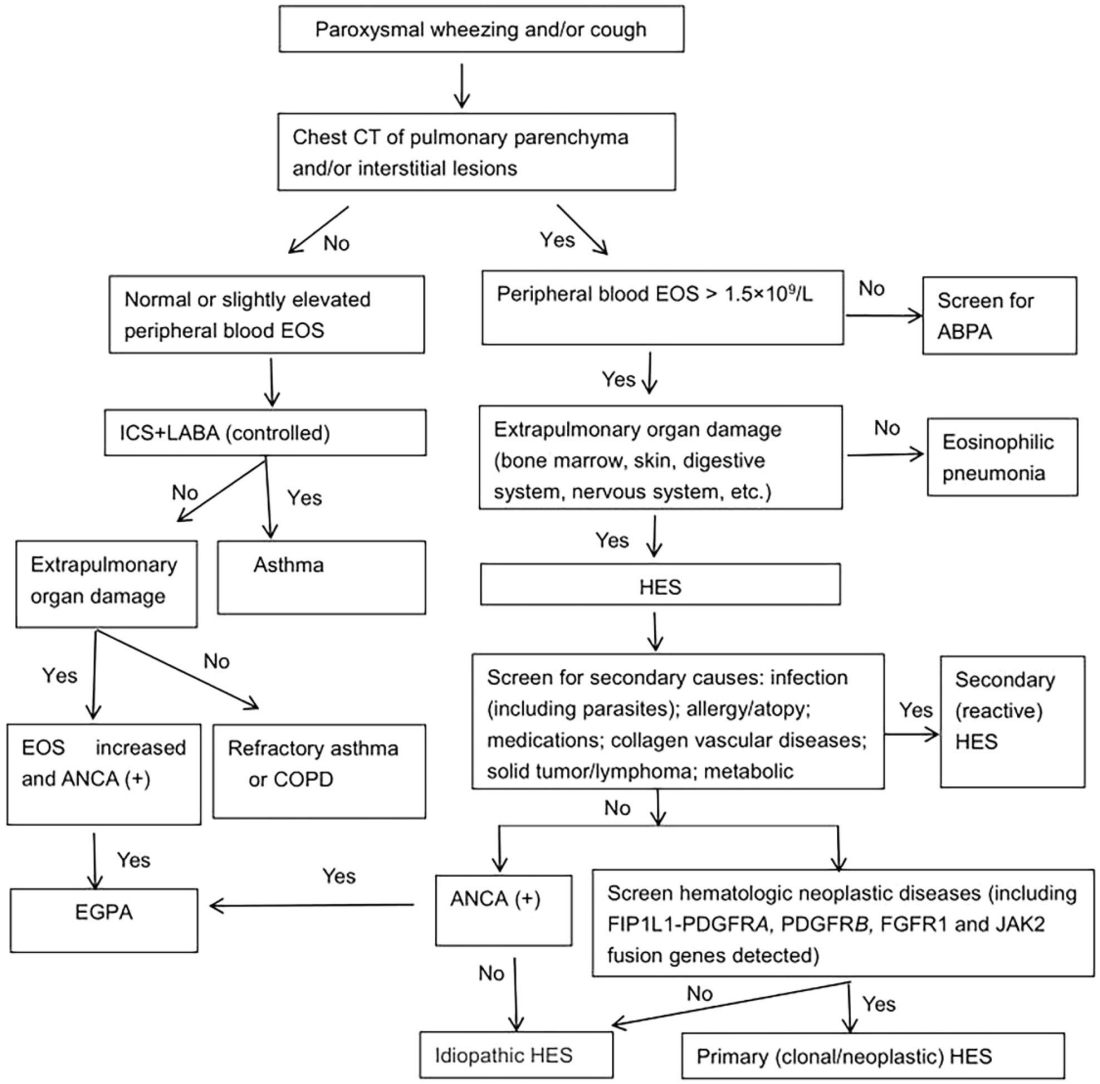
Diagnostic flow chart of asthma-like symptoms.

Repeated symptoms, signs of organ damage, and/or a significant increase in EOS at prednisone >10 mg/day indicate that treatment should be combined with other immunosuppressive therapies [27]. Hydroxyurea can be used as a first-line drug or in combination in steroid-insensitive patients, while interferon-α is usually used as a second-line agent after steroid treatment failure [26]. In recent years, TKIs have been applied to treat HES, which mainly depends on the aetiology and subtype and has strict indications. For example, imatinib is considered a definitive treatment for patients with HES in myeloid neoplasms (usually MDS/MPNs) and FIP1L1-PDGFRA rearrangement or PDGFRA/B-re-arranged neoplasms [29]. Imatinib was chosen when idiopathic HES did not respond or was not applicable to corticosteroids therapy [30]. Fortunately, our patient showed a good response to corticosteroids therapy. In addition, case reports have found that alemtuzumab (anti-CD52 monoclonal antibody) had a certain effect on refractory HES [31]. In addition, anti-IL-5 and anti-IL-5 receptors for HES treatment are still in the research stage, and several drugs have reached phase 2 and phase 3 trials [26]. Idiopathic HES is not only an exclusive but also a temporary diagnosis. It has been reported that patients developed hematological malignant tumours 10 years later [32]. For patients who are still prone to recurrent or severe systemic damage after therapy, long-term follow-up and regular review of autoantibodies is required. Based on the experience of this study, a diagnostic flowchart for asthma-like symptoms is presented, which may help reduce misdiagnosis (Figure 3).

## Conclusions

In summary, asthma-like symptoms, obstructive ventilatory disorder or positive BDT, and poor response to ICS are not necessarily indicative of refractory asthma; patients with EOS count >1.5 × 10^9^/L should be considered for the possibility of HES. The clinical characteristics of HES of different aetiologies are similar. Systemic corticosteroid therapy is preferred for both idiopathic and parasitic infections, the latter requiring combined deinsectization and showing good clinical efficacy. Idiopathic HES is treated with prolonged corticosteroids and relapses easily.

## Data Availability

The authors confirm that the data supporting the findings of this study are available within the article and its supplementary materials.

## Abbreviations

HES: Hypereosinophilic syndrome
EOS: eosinophils
HES_US_: idiopathic hypereosinophilic syndrome
HES_N_: primary hypereosinophilic syndrome
HES_R_: secondary hypereosinophilic syndrome
AHR: airway hyperresponsiveness
ECG: electrocardiography
IgE: immunoglobulin E
BDT: bronchial dilation test
CT: computed tomography
COPD: chronic obstructive pulmonary disease
BALF: bronchoalveolar lavage fluid
ICS: inhaled corticosteroids
ABPA: Allergic bronchopulmonary aspergillosis
